# Supplementary material for: Complete Mitogenome and Phylogenetic Analysis of the Carthamus tinctorius L
Source: Genes (Basel). 2023 Apr 26;14(5):979. doi: 10.3390/genes14050979 (PMC10217954; doi:10.3390/genes14050979)
Supplement: Supplementary file 1 [file genes-14-00979-s001.zip › genes-2325741-supplementary/Figure S1.pdf]

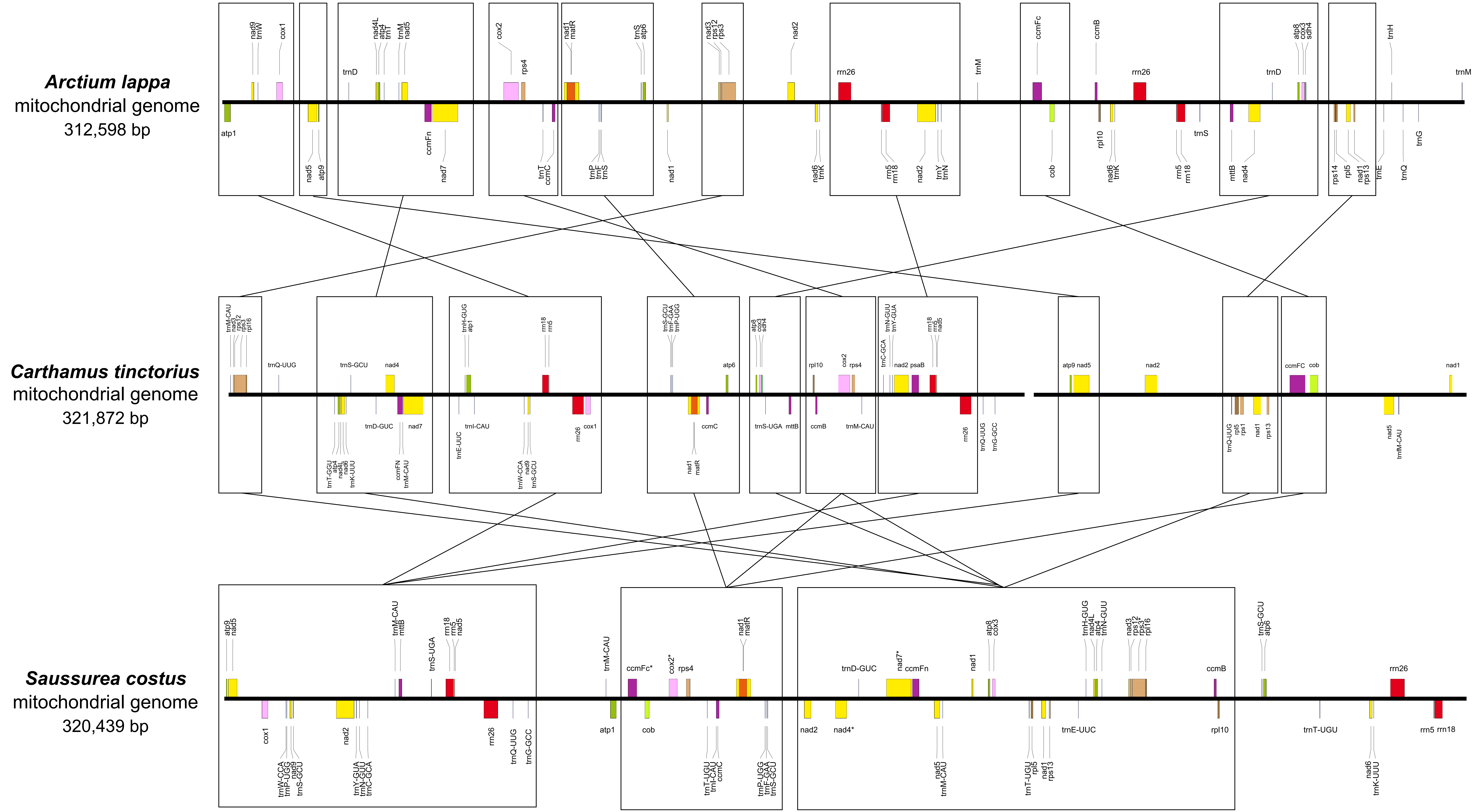

Arctium lappa
mitochondrial genome
312,598 bp
Carthamus tinctorius
mitochondrial genome
321,872 bp
Saussurea costus
mitochondrial genome
320,439 bp
