# Supplementary figures and images for: Complete Mitogenome and Phylogenetic Analysis of the Carthamus tinctorius L
Source: Genes (Basel). 2023 Apr 26;14(5):979. doi: 10.3390/genes14050979 (PMC10217954; doi:10.3390/genes14050979)

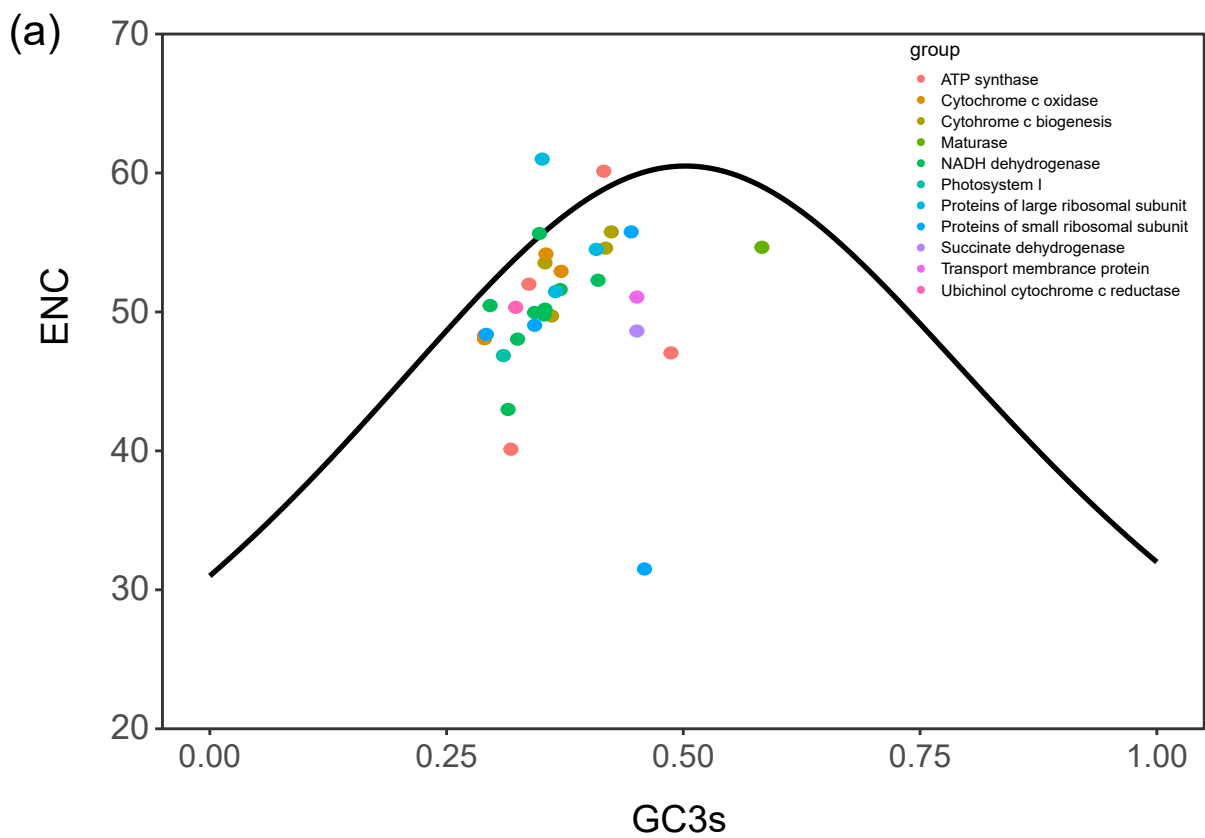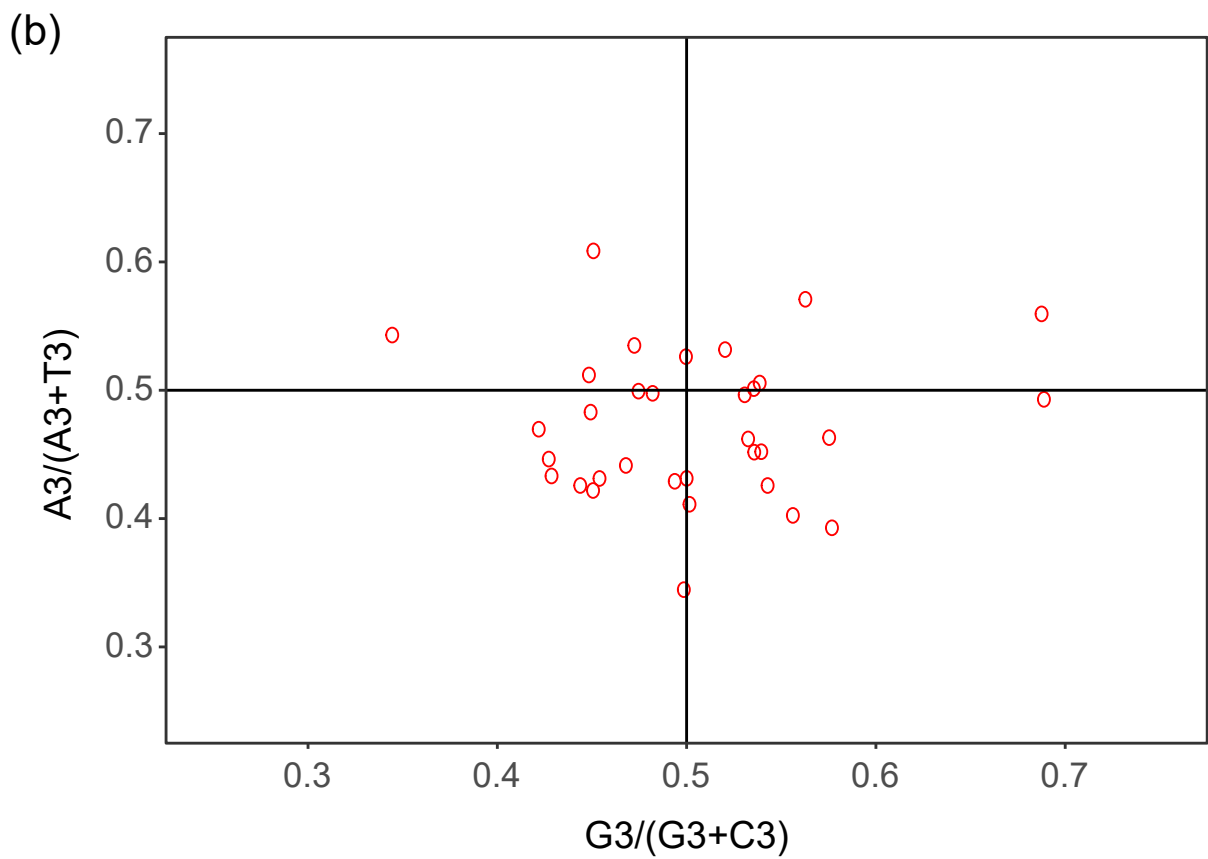

Supplement: Supplementary file 1 [file genes-14-00979-s001.zip › genes-2325741-supplementary/Figure S2.pdf]
